# Real-world treatment patterns and survival in extensive stage small-cell lung cancer in Japan

**DOI:** 10.1093/jjco/hyae175

**Published:** 2024-12-20

**Authors:** Hidehito Horinouchi, Chia-Hsien Suzu Chang, Jaime Shaw, Olga Archangelidi, Akhila Balasubramanian, Xerxes Pundole

**Affiliations:** Department of Thoracic Oncology, National Cancer Center Hospital, 5-1-1 Tsukiji, Chuo-ku, Tokyo 104-0045, Japan; Center for Observational Research, Amgen K.K., 9-7-1 Akasaka, Minato-ku, Tokyo 107-6239, Japan; Center for Observational Research, Amgen Inc., One Amgen Center Drive, Thousand Oaks, CA 91320, United States; Center for Observational Research, Amgen Ltd., 4 Uxbridge Business Park, Sanderson Road, Uxbridge UB8 1DH, United Kingdom; Center for Observational Research, Amgen Inc., One Amgen Center Drive, Thousand Oaks, CA 91320, United States; Center for Observational Research, Amgen Inc., One Amgen Center Drive, Thousand Oaks, CA 91320, United States

**Keywords:** extensive stage, small-cell lung cancer, retrospective, anti-PD-L1 therapy, overall survival

## Abstract

**Objective:**

To describe standard of care and inform the evolving unmet need among extensive stage small-cell lung cancer (ES-SCLC) patients in Japan since approval of first-line anti-PD-L1 therapies, we describe treatment patterns and overall survival by line of therapy.

**Methods:**

We conducted a descriptive analysis of adult ES-SCLC patients in Japan using de-identified patient data within the MDV database (hospital-based claims) to describe treatment patterns and DeSC database (payer-based claims linked to mortality of municipality records) to describe both treatment patterns and real-world overall survival (rwOS).

**Results:**

The study population of MDV and DeSC cohorts included 6302 and 903 patients, respectively. First-line anti-PD-L1 therapy-based regimens grew since their approval in 2019 and were used in ~35% and ~59% of patients in 2022, in the MDV and DeSC cohorts, respectively. Amrubicin monotherapy was the most common second-line (2 L) regimen before and after 1 L anti-PD-L1 approvals. No clear standard of care was identified in third-line (3 L) and fourth-line (4 L). Median rwOS following 1 L therapy was 10.6 months (95% CI: 9.0, 11.8) and 9.3 months (95% CI: 8.3, 10.3) in patients who did and did not receive anti-PD-L1 therapy, respectively. Following 2 L, 3 L, and 4 L therapy, median rwOS was 6.7 months (95% CI: 5.9, 7.4), 5.5 months (95% CI: 4.4, 6.4), and 4.7 months (95% CI: 3.4, 6.9), respectively.

**Conclusions:**

Anti-PD-L1 therapies have become part of first-line standard of care but survival in treated Japanese ES-SCLC patients remains poor, highlighting the unmet medical need in the post anti-PD-L1 era.

## Teaser

Study analyzed ES-SCLC treatment patterns and survival in Japan using MDV and DeSC databases. Anti-PD-L1 therapies are now first-line, but survival remains poor, highlighting unmet medical needs.

## Introduction

Small-cell lung cancer (SCLC) is an aggressive high-grade neuroendocrine tumor that accounts for ~10%–15% of all lung cancer [1, 2]. SCLC patients have a poor prognosis with 5-year overall survival of <10% [3]. After nearly two decades of limited change in standard of care, anti-programmed death-ligand 1 (anti-PD-L1) immunotherapies (atezolizumab or durvalumab), in combination with platinum-based therapy (carboplatin or cisplatin), received regulatory approval in Japan (atezolizumab in 2019 and durvalumab in 2020) as a first-line treatment option for patients with extensive stage (ES) SCLC. These anti-PD-L1 therapies were subsequently included in the Japan Lung Cancer Society treatment guidelines [4]. Addition of atezolizumab or durvalumab to platinum-based chemotherapy regimen in the first-line ES-SCLC setting demonstrated improvement in overall survival by about 2 months from 10.3–10.5 to 12.3–12.9 months in the clinical trials [5–8].

Real-world data on treatment patterns and outcomes in Japan following the approvals of anti-PD-L1 therapy are sparse or of limited sample size from a single institution [9–12]. To describe the evolving treatment paradigm and inform addressing the unmet need among ES-SCLC patients in Japan since approval of first-line anti-PD-L1 therapies, we conducted an observational study to describe real-world treatment patterns and overall survival by line of therapy in the Japanese ES-SCLC population.

## Patients and methods

### Data sources

We used two claims-based data sources. The MDV (Medical Data Vision Co., Ltd) hospital-based claims database and DeSC (DeSC Healthcare Co., Ltd.) payer-based claims database. Given the unique characteristics of each database, MDV was used to describe treatment patterns and DeSC was used to describe treatment patterns and to estimate real-world overall survival (rwOS). MDV includes ~20% of all oncology care in Japan, including >200 cancer centers. MDV contains demographic data, clinical data (stage, smoking, metastasis), tumor data (histology), and treatment information. Mortality in MDV is incomplete and not reported here. DeSC includes ~8.3 million Japanese people of all ages enrolled in public or private health insurance. DeSC contains demographic data, clinical data (smoking, metastasis), tumor data (histology), treatment information, and mortality data. Mortality data include deaths captured in-hospital and outside of hospitals via linkage to municipality records. The completeness of mortality data is an estimated 98%, based on the reported deaths from municipalities compared with those documented in the centralized vital statistics [13].

### Ethics approval

A waiver of ethical approval was received for this retrospective analysis of secondary data. We obtained the exemption certificate from the Institutional Review Board after submitting for their review on 17 August 2022 (PHRF-IRB-22H0004) and on 4 December 2023 (PHRF-IRB-23 L0004). All data were de-identified and handled in compliance with the Japanese Ethical Guidelines for Medical and Health Research Involving Human Subjects. As the data were de-identified for secondary use, the overlap between the MDV and DeSC databases is unknown.

### Study population

The two study cohorts (MDV and DeSC) included adult patients diagnosed with ES-SCLC based on the presence of lung cancer International Classification of Diseases [ICD]-10 code, C34X, and small-cell carcinoma specific disease code. The definitions used to identify ES-SCLC patients differed in each dataset. In the MDV cohort, patients were classified as ES-SCLC using AJCC eighth UICC TNM edition stages IIIB/C and IV as a proxy. In the DeSC cohort, patients were classified as ES-SCLC using secondary malignancy ICD-10 codes within 30 days before or after SCLC diagnosis, as a proxy for stage IV and ES-SCLC. The MDV cohort included ES-SCLC patients who initiated first line (1 L) of systemic anti-cancer therapy between 1 January 2016 and 31 December 2022. The DeSC cohort included ES-SCLC patients who initiated first line of systemic anti-cancer therapy between 1 April 2015 and 31 August 2022.

To increase likelihood of capturing the entire patient treatment journey from incident SCLC diagnosis, patients were excluded if (i) they received any systemic anti-cancer therapy between 14 and 365 days prior to SCLC diagnosis or (ii) if there was a gap greater than 90 days between initial SCLC diagnosis and initiation of treatment. In MDV, patients were also excluded for (i) data suggestive of uncertainty in incident SCLC case definition defined by another prior lung cancer diagnosis or (ii) patients with unknown stage. In DeSC, patients were also excluded for (i) data suggestive of uncertainty in incident SCLC case definition defined by another prior lung cancer diagnosis, (ii) data suggestive of another prior primary or secondary malignancy, or (iii) no available linkage to municipality records for mortality capture.

### Study period

For both cohorts, data were available for at least 1 year prior to cohort entry (i.e. 1 January 2015 for the MDV cohort and 1 April 2014 for the DeSC cohort). For the MDV cohort, no minimum follow-up period was required as patient survival was not described for this cohort. Treatment patterns were described using all available data until the last medical claims date or the end of the study date for this cohort (i.e. 31 December 2022), whichever occurred earlier. In the DeSC cohort, the end of study was defined as 31 August 2023, allowing for a minimum of 12 months opportunity of potential follow-up to capture death events.

### Study variables

Demographic and clinical characteristics were captured at baseline, defined as 30 days before or after SCLC diagnosis. Lines of therapy were derived using oncologist defined rule-based criteria, using claims of each systemic antineoplastic agent. To describe treatment patterns, we grouped therapies into platinum-based regimen with immunotherapy (atezolizumab or durvalumab), platinum-based regimen without immunotherapy, or single agent chemotherapy (e.g. amrubicin, topotecan, etoposide, irinotecan, or paclitaxel). Codes used to define study data elements are listed in Supplemental Table 1.

The DeSC cohort was used to describe treatment patterns, as well as rwOS, defined as the time from start date of a given line of therapy to the date of death for an individual patient. Patients with no reported death event were right censored at their date of disenrollment from their insurance plan; or, in the absence of disenrollment date, at the date of the last insurance claim or end of study date, whichever was earlier.

### Statistical analysis

Baseline disease characteristics were presented as frequency (percentage) for categorical data and as mean (standard deviation [SD]) or median (range: minimum, maximum) for continuous data. Missing and unknown values were not imputed and were included in the calculation of percentages. Distribution of treatments was described by line of therapy and by time period. In addition, sankey diagrams were used to illustrate treatment patterns in the MDV cohort. A sensitivity analysis was conducted to describe the use of anti-PD-L1 regimens in 1 L over time in the subset of patients in the MDV cohort who had stage IV at initial SCLC diagnosis, to enable comparisons in treatment patterns across datasets (MDV and DeSC).

Median rwOS was estimated by line of therapy using the Kaplan–Meier method. The estimated survival probabilities for rwOS and corresponding 95% CIs were estimated at 3-, 6-, 12-, and 24-month timepoints. We estimated rwOS by each line of therapy in the overall DeSC study cohort and in subsets defined based on the use of first-line therapy with or without an anti-PD-L1 agent. All analyses were performed using SAS 9.4 (SAS Institute, Cary, NC, USA.)

## Results

### Baseline patient characteristics

**Figure 1:**
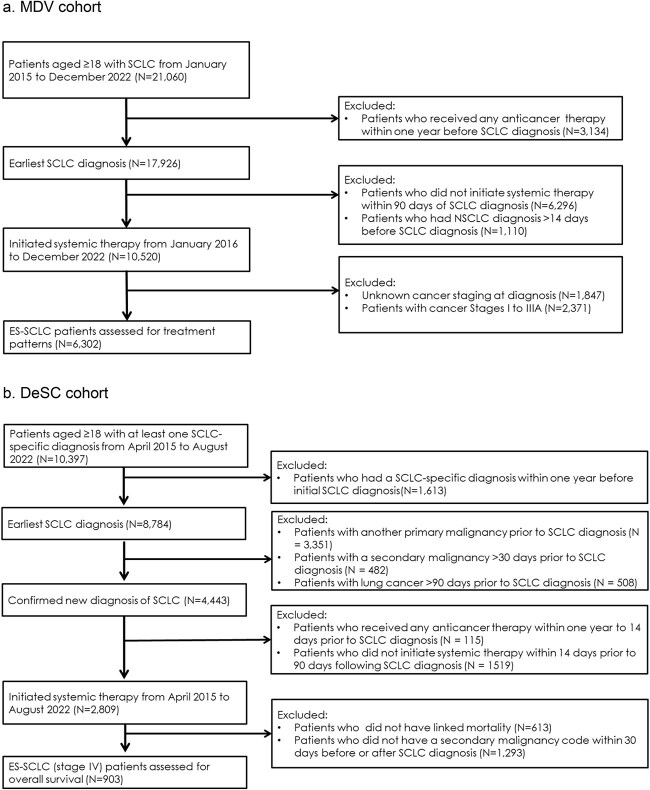
Patient attrition and flow. 1a. MDV cohort. b. DeSC cohort.

The study population included 6302 ES-SCLC patients in the MDV cohort and 903 ES-SCLC patients in the DeSC cohort who met the eligibility criteria (Fig. 1). The MDV cohort was of mean age 71 years and 81% were men. The DeSC cohort was of mean age 74 years and 82% were men. In the MDV cohort, where smoking status is available, 13% of patients had no smoking history. Approximately one-half of the ES-SCLC patients in the study cohorts were diagnosed in 2020 or later since the availability of immunotherapy (Table 1).

**Table 1:** Baseline characteristics among extensive stage small-cell lung cancer patients in MDV and DeSC cohorts.

	**MDV** ***n* (%)**	**DeSC** ***n* (%)**
***N***	6302	903
**Age**		
Age, mean (SD)	71.3 (8.0)	73.9 (6.8)
Age group, n (%)		
< 64 years	1091 (17.3)	76 (8.4)
65–74 years	2960 (47.0)	334 (37.0)
75+ years	2251 (35.7)	493 (54.6)
**Gender**		
Male	5102 (81.0)	741 (82.1)
Female	1200 (19.0)	162 (17.9)
**Type of insurance**		
Public health insurance	NA	401 (44.4)
Late-stage elderly heath system	NA	502 (55.6)
**Year of initial SCLC diagnosis**		
2015–2019	3785 (60.1)	441 (48.8)
2020–2022	2517 (39.9)	462 (51.2)
**Smoking status**		
Yes	4944 (78.5)	85 (9.4)
No	811 (12.9)	76 (8.4)
Missing/Unknown	547 (8.7)	742 (82.2)

### Treatment characteristics by line of therapy in the MDV and DeSC cohorts

**Figure 2:**
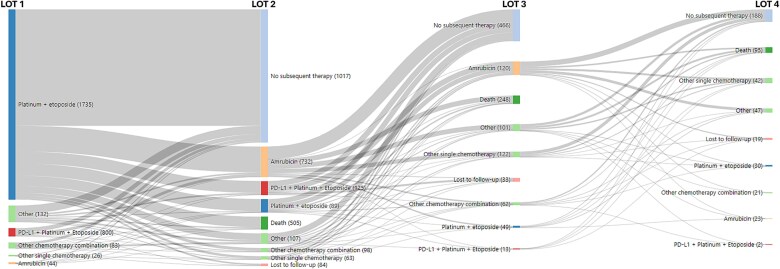
The flow of patients through regimens of therapy among extensive stage small-cell lung cancer patients in MDV cohort initiating therapy from 2019 to 2022.

In the MDV cohort of patients initiating 1 L therapy (*n* = 6302), 51% (3241/6302) received 2 L therapy, 46% (1480/3241) of 2 L-treated patients received 3 L therapy, and 41% (602/1480) of 3 L-treated patients received 4 L therapy. In the time period (≥2020) after anti-PD-L1 approval, platinum + etoposide without anti-PD-1 L agents were the most frequently used 1 L regimens (*n* = 1802; ~64%), followed by platinum + etoposide with anti-PD-L1 agents (*n* = 817; 29%). In 1 L, platinum and irinotecan-based regimens were used more often (18%) prior to approval of anti-PD-L1 inhibitors than after their approval (~2%). In the second line (2 L) setting, patients most frequently received amrubicin monotherapy (overall *n* = 1934; 60%) before and after approval of anti-PD-L1 therapies. In the third-line (3 L) and fourth-line settings, no clear standard of care was identified, and most patients received single agent chemotherapeutic regimens over combination therapy (Table 2). The visualization of the treatment sequence after anti-PD-L1 approvals illustrates the adoption of immunotherapies, which have become part of the treatment paradigm in ES-SCLC (Fig. 2).

**Table 2:** Use of treatments among patients with extensive stage small-cell lung cancer in the MDV and DeSC cohorts, by line of therapy and time period before and after approval of first-line anti-PD-L1 therapy.

	**MDV cohort**			**DeSC cohort**		
	**Overall** ***n* (%)**	**≤2019** ***n* (%)**	**≥2020** ***n* (%)**	**Overall** ***n* (%)**	**≤2019** ***n* (%)**	**≥2020** ***n* (%)**
**1 L**						
N	6302	3482	2820	903	436	467
Platinum + Etoposide + no PD-L1	4411 (70.0)	2609 (74.9)	1802 (63.9)	532 (58.9)	339 (77.8)	193 (41.3)
Platinum + Etoposide + PD-L1	818 (13.0)	1 (0.0)	817 (29.0)	264 (29.2)	18 (4.1)	246 (52.7)
Platinum + Irinotecan	705 (11.2)	641 (18.4)	64 (2.3)	63 (7.0)	56 (12.8)	7 (1.5)
Amrubicin monotherapy	127 (2.0)	83 (2.4)	44 (2.3)	4 (0.4)	1 (0.2)	3 (0.6)
Topotecan monotherapy	12 (0.2)	10 (0.3)	2 (0.1)	2 (0.2)	2 (0.5)	–
Taxane monotherapy	9 (0.1)	7 (0.2)	2 (0.1)	1 (0.1)	1 (0.2)	–
Other single-agent chemotherapy	109 (1.7)	62 (1.8)	47 (1.7)	6 (0.7)	4 (0.9)	2 (0.4)
All other regimens	111 (1.8)	69 (2.0)	42 (1.6)	31 (3.4)	15 (3.4)	16 (3.4)
**2 L**						
N	3241	2027	1214	454	175	279
Platinum + Etoposide + no PD-L1	384 (11.9)	293 (14.5)	91 (7.5)	36 (7.9)	23 (13.1)	13 (4.7)
Platinum + Etoposide + PD-L1	181 (5.6)	55 (2.7)	126 (10.4)	17 (3.7)	3 (1.7)	14 (5.0)
Platinum + Irinotecan	175 (5.4)	130 (6.4)	45 (3.7)	7 (1.5)	4 (2.3)	3 (1.1)
Amrubicin monotherapy	1934 (59.7)	1202 (59.3)	732 (60.3)	312 (68.7)	108 (61.7)	204 (73.1)
Topotecan monotherapy	206 (6.4)	123 (6.1)	83 (6.8)	21 (4.6)	5 (2.9)	16 (5.7)
Taxane monotherapy	56 (1.7)	31 (1.5)	25 (2.1)	13 (2.9)	4 (2.3)	9 (3.2)
Other single-agent chemotherapy	144 (4.4)	105 (5.2)	39 (3.2)	20 (4.4)	12 (6.9)	8 (2.9)
All other regimens	161 (5.0)	88 (4.4)	73 (6.0)	28 (6.2)	16 (9.1)	12 (4.3)
**3 L**						
N	1480	1013	467	193	72	121
Platinum + Etoposide + no PD-L1	197 (13.3)	147 (14.5)	50 (10.7)	19 (9.8)	9 (12.5)	10 (8.3)
Platinum + Etoposide + PD-L1	53 (3.6)	40 (4.0)	13 (2.8)	2 (1.0)	2 (2.8)	–
Platinum + Irinotecan	123 (8.3)	75 (7.4)	48 (10.3)	19 (9.8)	5 (6.9)	14 (11.6)
Amrubicin monotherapy	437 (29.5)	317 (31.3)	120 (25.8)	43 (22.3)	19 (26.4)	24 (19.8)
Topotecan monotherapy	230 (15.5)	146 (14.4)	84 (18.0)	28 (14.5)	6 (8.3)	22 (18.2)
Taxane monotherapy	29 (6.1)	21 (2.1)	8 (1.7)	11 (5.7)	6 (8.3)	5 (4.1)
Other single-agent chemotherapy	321 (21.7)	206 (20.3)	115 (24.6)	60 (31.1)	20 (27.8)	40 (33.1)
All other regimens	90 (6.1)	61 (6.0)	29 (6.2)	11 (5.7)	5 (6.9)	6 (5.0)
**4 L**						
* N*	602	437	165	70	24	46
Platinum + Etoposide + no PD-L1	86 (14.3)	56 (12.8)	30 (18.2)	9 (12.9)	2 (8.3)	7 (15.2)
Platinum + Etoposide + PD-L1	16 (2.7)	14 (3.2)	2 (1.2)	0 (0.0)	–	–
Platinum + Irinotecan	41 (6.8)	32 (7.3)	9 (5.5)	2 (2.9)	1 (4.2)	1 (2.2)
Amrubicin monotherapy	91 (15.1)	68 (15.6)	23 (13.9)	7 (10.0)	1 (4.2)	6 (13.0)
Topotecan monotherapy	160 (26.6)	117 (26.8)	43 (26.1)	19 (27.1)	7 (29.2)	12 (26.1)
Taxane monotherapy	35 (5.8)	23 (5.3)	12 (7.3)	12 (17.1)	4 (16.7)	8 (17.4)
Other single-agent chemotherapy	108 (17.9)	78 (17.9)	30 (18.2)	16 (22.9)	7 (29.2)	9 (19.6)
All other regimens	65 (10.8)	49 (11.2)	165 (9.7)	5 (7.1)	2 (8.3)	3 (6.5)

1 L, first line; 2 L, second line; 3 L, third line; 4 L, fourth line.

In the DeSC cohort of the 903 total patients initiating 1 L therapy for ES-SCLC, therapy transition rates from 1 L to 2 L, 2 L to 3 L, and 3 L to 4 L were 50%, 42%, and 36%, respectively. In the time period (≥2020) after anti-PD-L1 approval, platinum + etoposide with anti-PD-L1 agents were the most frequently used 1 L regimens (*n* = 246; ~53%), followed by platinum + etoposide without anti-PD-L1 agents (*n* = 193; 41%). Other treatment characteristics for the DeSC cohort, by line of therapy, were mostly similar to those in the MDV cohort. Distribution of regimens used by line of therapy before (≤2019) and after (≥2020) anti-PD-L1 approvals in MDV and DeSC are outlined in Table 2.

### First-line use of anti-PD-L1 therapies over time in the MDV and DeSC cohorts

In the MDV cohort, the use of anti-PD-L1 therapies (atezolizumab or durvalumab) in combination with platinum-based regimens began in 2019 (6.4%) and increased to 24.3%, 31.0%, and 33.9% in 2020, 2021, and 2022, respectively. Atezolizumab was the most common anti-PD-L1 therapy in earlier years and durvalumab was the most common anti-PD-L1 therapy utilized in 2022. A similar increasing trend in the use of anti-PD-L1 therapies (atezolizumab or durvalumab) in combination with platinum-based regimens was observed in a sensitivity analysis that evaluated trends in treatment, in a subset of patients who had stage IV at initial SCLC diagnosis (Supplemental Table 2). Use of anti-PD-L1 therapies was 7.8% in 2019 and increased to 31.6%, 37.3%, and 40.7% in 2020, 2021, and 2022, respectively.

In the DeSC cohort, where all patients had metastasis at diagnosis, the use of anti-PD-L1 therapies combined with platinum-based regimen was 11.5% in 2019, increasing to 50.6%, 52.2%, and 58.5% in 2020, 2021, and 2022, respectively. Atezolizumab was the predominant anti-PD-L1 therapy in 2019 and 2020, but durvalumab was the most commonly used anti-PD-L1 therapy in 2021 and 2022. Details on use by specific regimens over time are described in Table 3.

**Table 3:** Use of first-line anti-PD-L1 therapy (atezolizumab or durvalumab) among extensive stage small-cell lung cancer patients over time in the MDV and DeSC cohorts.

**Year of 1 L regimen initiation**	**<2019** ***n* (%)**	**2019** ***n* (%)**	**2020** ***n* (%)**	**2021** ***n* (%)**	**2022** ***n* (%)**
**MDV cohort**					
***N***	2707	1070	925	878	722
**Any use of 1 L anti-PD-L1 regimens**					
**1 L regimens including anti-PD-L1**	0 (0.0)	68 (6.4)	225 (24.3)	272 (31.0)	245 (33.9)
atezolizumab + carboplatin + etoposide	0 (0.0)	68 (6.4)	198 (21.4)	147 (16.7)	110 (15.2)
carboplatin + durvalumab + etoposide	0 (0.0)	0 (0.0)	18 (1.9)	90 (10.3)	104 (14.4)
cisplatin + durvalumab + etoposide	0 (0.0)	0 (0.0)	6 (0.6)	31 (3.5)	28 (3.9)
carboplatin/cisplatin + durvalumab + etoposide	0 (0.0)	0 (0.0)	3 (0.3)	4 (0.5)	3 (0.4)
**DeSC cohort**					
***N***	280	156	164	180	123
**Any use of 1 L anti-PD-L1 regimens**					
**1 L regimens including anti-PD-L1** ^a^	0 (0.0)	18 (11.5)	83 (50.6)	94 (52.2)	72 (58.5)
atezolizumab + carboplatin + etoposide	0 (0.0)	18 (11.5)	73 (44.5)	44 (24.4)	32 (26.0)
carboplatin + durvalumab + etoposide	0 (0.0)	0 (0.0)	8 (4.9)	37 (20.6)	35 (28.5)
cisplatin + durvalumab + etoposide	0 (0.0)	0 (0.0)	2(1.2)	9 (5.0)	4 (3.3)
carboplatin/cisplatin + durvalumab + etoposide	0 (0.0)	0 (0.0)	0 (0.0)	1 (0.6)	0 (0.0)

1 L, first line; 2 L, second line; 3 L, third line; 4 L, fourth line.

^a^Total includes the use of anti-PD-L1 (atezolizumab or durvalumab) combined with therapies not listed in the table.

### Real-world overall survival of ES-SCLC patients in the DeSC cohort

Median rwOS estimates by line of therapy were 9.6 months (*n* = 903; 95% CI: 8.9, 10.3) following initiation of 1 L therapy, 6.7 months (*n* = 454; 95% CI: 5.9, 7.4) following the initiation of 2 L therapy, 5.5 months (*n* = 193; 95% CI: 4.4, 6.4) following the initiation of 3 L therapy, and 4.7 months (*n* = 70; 95% CI: 3.4, 6.9) following the initiation of 4 L therapy (Table 4). Median rwOS was numerically higher in patients who received anti-PD-L1 therapy in 1 L (10.6 months [95% CI: 9.0, 11.8]; *n* = 267) compared with patients who did not receive anti-PD-L1 therapy in 1 L (9.3 months [95% CI: 8.3, 10.3]; *n* = 636). Median rwOS estimates by 1 L anti-PD-L1 receipt were numerically similar in patients who received 2 L, 3 L, and 4 L. Survival probability estimates at 3, 6, 12, and 24 months following each respective (1 L, 2 L, 3 L, or 4 L) line of therapy initiation are provided in Table 4.

**Table 4:** Real-world overall survival among extensive stage small-cell lung cancer patients in Japan, by line of therapy and first-line anti-PD-L1 use in the DeSC cohort.

	**n/N**	**Median rwOS, months (95% CI)**	**3-months rwOS % (95% CI)**	**6-months rwOS % (95% CI)**	**12-months rwOS % (95% CI)**	**24-months rwOS % (95% CI)**
**Overall cohort**						
1 L	732/903	9.6 (8.9, 10.3)	85.0 (82.5, 87.2)	68.1 (64.9, 71.1)	38.7 (35.4, 42.0)	13.5 (11.1, 16.2)
2 L	376/454	6.7 (5.9, 7.4)	77.1 (72.9, 80.7)	54.1 (49.3, 58.7)	27.5 (23.1, 32.0)	8.6 (5.8, 12.0)
3 L	168/193	5.5 (4.4, 6.4)	73.7 (66.7, 79.4)	46.0 (38.6, 53.1)	17.1 (11.8, 23.2)	4.3 (1.8, 8.6)
4 L	64/70	4.7 (3.4, 6.9)	67.7 (55.3, 77.4)	42.2 (30.3, 53.6)	13.4 (6.4, 23.0)	2.2 (0.5, 27.7)
**Subset of patients in the overall cohort who received first-line regimen with an anti-PD-L1 agent**						
1 L	190/267	10.6 (9.0, 11.8)	90.2 (85.9, 93.2)	72.0 (66.1, 77.0)	42.8 (36.5, 49.0)	13.3 (8.4, 19.3)
2 L	104/146	6.9 (5.3, 8.2)	76.0 (68.0, 82.2)	53.6 (44.7, 61.8)	26.8 (18.8, 35.3)	11.8 (6.1, 19.5)
3 L	38/53	5.5 (4.3, 7.4)	79.9 (65.7, 88.7)	46.3 (31.0, 60.3)	11.2 (3.3, 24.5)	0.0 (.,.)
4 L	16/18	3.2 (1.3, 4.7)	52.9 (27.6, 73.0)	19.9 (5.0, 41.9)	6.6 (0.4, 25.8)	0.0 (.,.)
**Subset of patients in the overall cohort who received first-line regimen without an anti-PD-L1 agent**						
1 L	542/636	9.3 (8.3, 10.3)	82.8 (79.6, 85.5)	66.5 (62.6, 70.1)	37.0 (33.1, 40.9)	13.4 (10.7, 16.5)
2 L	272/308	6.7 (5.8, 7.8)	77.6 (72.5, 81.9)	54.4 (48.6, 59.9)	27.8 (22.7, 33.1)	8.3 (5.3, 12.1)
3 L	130/140	5.5 (4.2, 6.4)	71.5 (63.2, 78.3)	45.7 (37.2, 53.8)	18.5 (12.4, 25.6)	5.1 (2.1, 10.1)
4 L	48/52	5.6 (3.8, 7.9)	72.6 (58.2, 82.8)	49.1 (34.8, 61.8)	15.4 (6.9, 27.0)	2.9 (0.3, 12.3)

1 L, first line; 2 L, second line; 3 L, third line; 4 L, fourth line; CI, confidence interval.

## Discussion

Results from this large real-world study of Japanese patients with ES-SCLC highlight the evolving ES-SCLC treatment landscape and the persisting unmet medical needs of ES-SCLC patients in Japan. Following the introduction of anti-PD-L1 therapies in 2019, their use has been gradually increasing. Across the two large, population-level data sources used in this study, between 35% and 59% of 1 L treated ES-SCLC patients received a platinum-based regimen with an anti-PD-L1 therapy by 2022. Approximately one-fifth of patients received a platinum- and irinotecan-based regimen in 1 L before the approval of anti-PD-L1 therapies; however, the use of this combination in 1 L decreased drastically following the approvals of 1 L anti-PD-L1 therapies, indicating a shift in the overall treatment paradigm. A small proportion of patients also received anti-PD-L1 therapy-based regimens in 2 L and 3 L.

Studies have reported the adoption rate of newly approved immunotherapy regimens for 1 L therapy in ES-SCLC in the EU [14] and the USA [15]. A study from Europe reported that, by 2021, anti-PD-L1 therapy-based regimens represented up to 40%–55% of first-line treatment for SCLC in the UK, France, and Germany, with lower rates (~20% to 30%) in Spain and Italy [14]. In a real-world study using data from a large oncology focused electronic health record-based system in the USA, a large majority (~80%) of 1 L treated patients in 2020 and 2021 received combination therapy with an anti-PD-L1 agent [15]. The average ages in the MDV and DeSC study cohorts were 71 and 74 years, respectively, compared with ~68 years in the US study; it is possible that the older ES-SCLC population in our study represented patients who were less fit to receive immunotherapy due to their underlying conditions. Differences in the recommendations for use of anti-PD-L1 therapies in SCLC treatment guidelines between USA, EU, and Japan may also partially explain the disparities in the use of first-line anti-PD-L1 therapies between the regions. SCLC treatment guidelines in the EU and Japan recommend use of anti-PD-L1 therapies in ES-SCLC patients with ECOG performance status ≤1 [4, 16], while no restriction related to ECOG performance status is applied in US guidelines [17]. In the US study by Shaw *et al*. [15], ~25% of the anti-PD-L1 treated patients had an ECOG performance score ≥2. The proportion of patients in Japan with ECOG performance ≥2 has been estimated to be between 10% and 30% [18–20], although ECOG information was not available in the MDV or DeSC datasets and firm conclusions related to the impact of ECOG performance on use of these therapies cannot be drawn. Furthermore, despite seeing a consistent trend in the increased uptake of anti PD-L1 therapies in 1 L treatment over time across both of our study cohorts, the prevalence of use was greater in the DeSC cohort. It may be that the proportion of patients deemed eligible for 1 L anti-PDL1 therapy was greater in this cohort, which was restricted to metastatic patients, compared with the broader MDV cohort of all ES-SCLC patients. A sensitivity analysis conducted in the MDV cohort restricting to patients with Stage IV metastatic disease at initial diagnosis showed a higher proportion of patients utilizing anti-PD-L1 therapies in combination with platinum-based regimens, when compared with all patients considered to be ES-SCLC (AJCC eighth UICC TNM edition stages IIIB/C and IV) in the MDV cohort. This sensitivity analysis evaluated treatment patterns in a cohort considered to be more specific for ES-SCLC disease and showed that ~41% of patients received anti-PD-L1 therapy in 1 L. Results from the DeSC and MDV cohorts suggest that stage impacts use of therapies. However, it does not completely explain away difference in the use of anti-PD-L1 therapies across cohorts, indicating the potential role of other clinical factors (not evaluable in this study) in the use of therapies. The use of anti-PD-L1 therapies in ES-SCLC may change over time as clinical experience and evidence from real-world studies continue to build.

Our findings also highlight the poor survival outcomes of ES-SCLC across lines of therapy. We found that median rwOS following first-line treatment was numerically higher in patients who received anti-PD-L1 therapies in 1 L compared with those who did not. This finding is directionally in alignment with clinical trial observations [5–8]. The shorter median OS observed in our real-world study cohort compared with clinical trials might be attributable to several clinical factors including older or more fragile patients, higher rates of comorbidities and metastases, and other poor prognostic factors in the real-world. However, the impact of these factors was not evaluable in the current analysis due to lack of relevant data elements in the databases. A prior Dutch study evaluating the efficacy-effectiveness gap in ES-SCLC patients showed a 21% shorter survival in real-world patients, compared with trial participants, which was at least partially attributable to differences in baseline clinical factors [21]. Survival outcomes among patients who did not receive anti-PD-L1 therapy in our study were generally consistent with older publications with data prior to the approvals of anti-PD-L1 therapies in Japan [20, 22, 23]. A few studies have reported on early experience with anti-PD-L1s in the real-world setting in Japan but are limited to a few institutions and small overall sample sizes [9–12,24–26]. In this study, patients who received 1 L anti-PD-L1 based therapy had median rwOS of 10.6 months (95% CI: 9.0, 11.8). In contrast, a few recent studies from Japan have reported median OS of 10.0 months (95% CI: 7.8, 16.1) to 20.8 months (95% CI: 13.9, 37.2) [9,24–27]. Differences in estimates between our study and others might be attributable to potential differences in baseline risk between the cohorts, including older age, differential distribution of gender, differences in staging, ECOG performance status (which was not evaluable in our study), and the small sample size of the cohorts investigated in the other real-world studies.

Stage is a critical prognostic factor impacting outcomes. Extensive stage in our study was defined based on the availability of secondary metastases at the time of SCLC diagnosis and, as such, may reflect a subset with potentially poorer prognosis, of all patients who may be considered ES in clinical practice. The Ano *et al*. [9], Ishida *et al*. [24], and Morimoto *et al*. [26] studies included patients with ES-SCLC at diagnosis, while those by Kubo *et al*. [25] (recurrence following LS-SCLC in ~14%) and Igata *et al*. [27] (unspecified staging at diagnosis) included recurrent patients, which may explain the differences observed in the median OS.

In the 2 L, 3 L, and 4 L treatment settings, we did not observe any differences in median OS among patients who were treated with and without an anti-PD-L1 inhibitor. Our findings are generally consistent by line of therapy with a recent publication using data from a large oncology focused electronic health record-based system of primarily community oncology practice-based patients in the USA [15].

Key strengths of our study include the use of two large data sources representative of Japanese patients receiving treatment for ES-SCLC. The MDV data network covers over 200 cancer centers across Japan using the Diagnosis Procedure Combination (DPC) system. Since cancer treatment is likely to be administered in DPC-designated hospitals, the results from this study should be generalizable to patients treated for SCLC in Japan. Sankey visualizations for treatment sequencing are provided for MDV cohort, which reflect oncology care in Japan. The use of claims-based data sources has traditionally been challenging due to the lack of codes needed to specifically identify SCLC patients [28]. Data sources leveraged in our study contained SCLC disease specific codes used in Japan, which have previously been validated in a similar data source in Japan [29]. We estimated rwOS using data from a large claims database with linked mortality information from municipality records.

Our study has a few limitations. Treatment patterns were evaluated based on derivation of line of therapy information, and were algorithmically defined based on *a priori* rules. Our findings were in keeping with treatment recommendations based on JLCS guidelines; however, misclassification of lines of therapy cannot be completely ruled out, due to the algorithmic nature of line of therapy derivation. Missing information is a key challenge in real-world data assessments. In our study we did not have access to ECOG performance status, thus limiting our ability to fully contextualize our findings. In addition, baseline comorbidities, metastases, and adverse events from systemic anti-cancer therapies can influence choice of anti-cancer therapy, as well as outcomes. These factors were not evaluable in our study due to lack of relevant data elements in the claims data sources. We used the DeSC database to estimate rwOS using linked mortality information, for patients with metastasis at SCLC diagnosis (a proxy for ES-SCLC). Our rwOS results for patients with metastasis at diagnosis are reflective of a majority of ES-SCLC patients. However, underestimation of rwOS in our study cannot be ruled out due to non-capture of potential ES-SCLC patients without metastasis at diagnosis.

In summary, results from our study describe the evolving ES-SCLC treatment landscape in Japan and highlight the poor outcomes observed in patients with ES-SCLC in the real-world setting, even in a post immunotherapy era. Future studies are needed to identify which patient groups are most likely to benefit from immunotherapy, allowing for better individualized treatment strategies.

## Conclusion

In this large and contemporaneous evaluation, our findings provide insights into the evolving standard of care in ES-SCLC. The use of anti-PD-L1 therapies in first line grew to one-third and two-thirds of the treated ES-SCLC population in 2022, in the MDV and DesC cohorts, respectively. However, survival outcomes across lines of therapy are poor despite the use of anti-PD-L1 therapies. Real-world assessments of the treatment landscape and outcomes, as described in our study, will inform the best clinical practices and shape continued clinical development to meet the unmet medical needs of patients with SCLC.

## Supplementary Material

Supplemental_material_fin_hyae175

## Data Availability

The data used in this study are provided by Medical Data Vision Co. Ltd. and DeSC Healthcare Co. Ltd. Due to licensing restrictions specific to this study, the data cannot be made publicly accessible.
