# Supplementary material for: Real-world treatment patterns and survival in extensive stage small-cell lung cancer in Japan
Source: Jpn J Clin Oncol. 2024 Dec 20;55(4):383–90. doi: 10.1093/jjco/hyae175 (PMC11973631; doi:10.1093/jjco/hyae175)
Supplement: Supplemental_material_fin_hyae175 [file supplemental_material_fin_hyae175.docx]

**9. Supplemental material**

**Table 1.** Codes used to define study data elements

| Diagnosis | Code type | Code | Applied data source |
| --- | --- | --- | --- |
| SCLC | Disease code | 8842185, 8847633, 8847594, 8847660, 8847675 | DeSC |
| NSCLC | Disease code | 8847678, 8847637, 8847664, 8847598, 8849238, 8847732, 8842053, 8847272 | MDV |
| All other cancers | ICD-10 codes | C00 – C26, C30 – C33, C37 – C58, C60 – C76, C80 – C86, C88 – C96, D37 – D44, D47 – D49 | DeSC |
| Metastases codes | Disease code | 1960005 , 8834006, 8842736, 8848703, 8845718, 8842764, 8832487, 8844689, 8839656, 8839655, 8845706, 8845660, 8848695, 8848689, 8848748, 8842679, 8836801, 8845709, 8844873, 8845584, 1969001, 1970006, 1970005, 1971002, 1972003, 8847850, 1972004, 8847463, 8847055, 8847793, 8844084, 8837929, 8837931, 8842810, 8842314, 8837926, 8839765, 1976001, 7895002, 2354005, 1977005, 1977006, 8837928, 8837930, 8837933, 1980004, 1981002, 8847798, 1982004, 1983019, 1983006, 1983009, 8835803, 1983025, 1921008, 1983014, 1984001 , 8842667, 8842788, 8843085, 8849346, 8849333, 8844349, 1985008, 8843072, 8849335, 8843088, 8842830, 8841259, 8843075, 8844442, 8846021, 8848241, 8849345, 8836022, 8837313, 8842692, 1985024, 1985007, 1985023, 1985025, 8842905, 8843015, 1985010, 1986005, 8837934, 1988009, 2398068, 4239002, 8833010, 8848981, 8843436, 8843435, 8845248, 8848470, 8848159, 1991019, 8837935, 1990009, 1990004 | DeSC |

| Treatment | Code type | Code | Applied data source |
| --- | --- | --- | --- |
| SCLC treatments (Docetaxel) | receiptcodes | 622435102, 622435002, 622408601, 622408501, 620919801, 622429401, 620919901, 622429301, 622068501, 622068601, 622354901, 622354801, 622295501, 622295601, 622285301, 622285201, 640411025, 622295001, 640411026, 622294901, 622285401, 622356501, 622356401, 622215301, 622215401, 622231901, 622231801, 622272101, 622272001, 622290401, 622290501, 622283101, 622283201, 622417701, 622417601 | MDV, DeSC |
| SCLC treatments (Cisplatin) | receiptcodes | 620923301, 620924101, 620004129, 620004131, 620008946, 620923701, 620006298, 620008948, 620006300, 620002591, 620001919, 620004130, 620923202, 620006299, 620008947, 620923602, 644290002, 620924002, 644290004, 620009547, 640406088, 620009545, 644290003, 640406090, 644210054, 640406089, 644210057, 622761000, 620009546, 622760800, 644210055, 644210052, 644210053, 644210056, 622760900, | MDV, DeSC |
| SCLC treatments (Carboplatin) | receiptcodes | 620004122, 620004732, 620007255, 620007256, 620007254, 620004734, 620004733, 621754702, 620004732, 621754602, 620004122, 621754502, 620004120, 620004119, 620004118, 620004121, 622098103, 622098203, 622098303, 620004117, 622882801, 622761100, 622882701, 622882601, 620000216, 622761300, 620004115, 620004114, 620002932, 620004116, 622761200 | MDV, DeSC |
| SCLC treatments (Etoposide) | receiptcodes | 620003643, 620008173, 620004777, 622220501, 620004760, 622101701, 620003642, 620006119, 620003643, 620006120, 622101703, 610406277, 622130501, 622903600 | MDV, DeSC |
| SCLC treatments (Paclitaxel) | receiptcodes | 620004170, 620004171, 620005689, 621970101, 622082101, 621970101, 620004171, 620004170, 622375101, 622375001, 620003752, 620003751, 620005689, 620005688, 622082101, 620005690, 622082001, 622259201, 622259101, 622760500, 622009101, 622009102, 622009202, 622009201, 622760700, 622760600 | MDV, DeSC |
| SCLC treatments (Amrubicin) | receiptcodes | 640462038, 640462039 | MDV, DeSC |
| SCLC treatments (Topotecan) | receiptcodes | 620005197 | MDV, DeSC |
| SCLC treatments (Irrinotecan) | receiptcodes | 620007257, 620009522, 644290008, 622091201, 622059801, 620009516, 620009521, 622808601, 622236901, 644290007, 622230201, 622019501, 622237001, 644290009, 620007258, 620919701, 622258901, 620009518, 622259001, 620009520, 621900302, 620009519, 622059701, 622019401, 622230301, 620009515, 620009517, 622091101, 620919501, 644290006, 621900402 | MDV, DeSC |
| SCLC treatments (Atezolizumab) | receiptcodes | 622594601, 622594601, 629900601 | MDV, DeSC |
| SCLC treatments (Durvalumab) | receiptcodes | 622633301, 622633201, 622633301 | MDV, DeSC |
| immune checkpoint inhibitors (Pembrolizumab) | receiptcodes | 622515801, 622515801, 622515701 | MDV, DeSC |
| immune checkpoint inhibitors (Nivolumab) | receiptcodes | 622364901, 622662201, 622364901, 622364801, 629911501 | MDV, DeSC |
| immune checkpoint inhibitors (Ipilimumab) | receiptcodes | 622440501, 629917301 | MDV, DeSC |
| Other treatments (Cyclophosphamide) | receiptcodes | 644210037, 640453101, 620005941, 622181601 | MDV, DeSC |
| Other treatments (Doxorubicin) | receiptcodes | 621983201, 621983301, 620003675, 621995301, 622014001, 621995401, 620004851 | MDV, DeSC |
| Other treatments (Gemcitabine) | receiptcodes | 621973501, 621973401, 640454012, 640454013, 622487801, 622028701, 622487701, 622028601, 622202501, 622202401, 622460401, 622019701, 622019601, 622460501, 621970301, 621970201, 622062203, 622062103, 622393101, 622393001, 621970302, 621994501, 621970202, 621994401, 22099001, 622098901, 622062205, 622062105, 622062201, 622062101, 622272801, 622272901 | MDV, DeSC |
| Other treatments (Temozolomide) | receiptcodes | 620004354, 622576901, 620004353, 622576801, 621982101 | MDV, DeSC |
| Other treatments (Vincristine) | receiptcodes | 640454006 | MDV, DeSC |
| Other Anti-cancer therapies | WHO ATC Code | \| L01 \| Antineoplastic Agent \| \| --- \| --- \| \| L02AA03 \| [ethinylestradiol (estrogen)](https://www.whocc.no/atc_ddd_index/?code=L02AA03) \| \| L02AB02 \| [medroxyprogesterone (progestogen)](https://www.whocc.no/atc_ddd_index/?code=L02AB02) \| \| L02AE03 \| [goserelin (Gonadotropin releasing hormone analogues)](https://www.whocc.no/atc_ddd_index/?code=L02AE03) \| \| L02BA01 \| [Anti-estrogens (endocrine therapy)](https://www.whocc.no/atc_ddd_index/?code=L02BA&showdescription=no) \| \| L02BA02 \| Endocrine therapy \| \| L02BA03 \| Endocrine therapy \| \| L02BB01 \| Endocrine therapy \| \| L02BB03 \| Endocrine therapy \| \| L02BB04 \| Endocrine therapy \| \| L02BG03 \| Endocrine therapy \| \| L02BG04 \| Endocrine therapy \| \| L02BG06 \| Endocrine therapy \| \| L02BX02 \| Endocrine therapy \| \| L02BX03 \| Endocrine therapy \| \| L03AX01 \| Immunostimulants \| \| L03AX03 \| Immunostimulants \| \| L04AX03 \| Immunosuppressants \| \| V03AF03 \| All other therapeutic products \| \| V03AF04 \| All other therapeutic products \| \| V03AF07 \| All other therapeutic products \| \| V10XX03 \| Therapeutic radiopharmaceuticals \| | DeSC |

Supplemental table 2. Use of first-line anti-PD-L1 therapy (atezolizumab or durvalumab) among small cell lung cancer patients diagnosed with stage IV disease, over time in the MDV cohort

| **Year of 1L regimen initiation** | **< 2019**  **n (%)** | **2019**  **n (%)** | **2020**  **n (%)** | **2021**  **n (%)** | **2022**  **n (%)** |
| --- | --- | --- | --- | --- | --- |
| **MDV cohort (subset with Stage IV at initial SCLC diagnosis)** |  |  |  |  |  |
| **N** | 2,173 | 783 | 630 | 648 | 560 |
| **Any use of 1L anti-PD-L1 regimens** |  |  |  |  |  |
| **1L regimens including anti-PD-L1** | 0 (0.0) | 61 (7.8) | 199 (31.6) | 242 (37.3) | 227 (40.7) |
| atezolizumab + carboplatin + etoposide | 0 (0.0) | 61 (7.8) | 174 (27.6) | 129 (19.9) | 102 (18.2) |
| carboplatin + durvalumab + etoposide | 0 (0.0) | 0 (0.0) | 16 (2.5) | 83 (12.8) | 97 (17.3) |
| cisplatin + durvalumab + etoposide | 0 (0.0) | 0 (0.0) | 6 (1.0) | 26 (4.0) | 21 (3.8) |
| carboplatin/cisplatin + durvalumab + etoposide | 0 (0.0) | 0 (0.0) | 3 (0.5) | 4 (0.6) | 7 (1.3) |
